# Tumor Microenvironment and Metabolism

**DOI:** 10.3390/ijms18122729

**Published:** 2017-12-16

**Authors:** Li V. Yang

**Affiliations:** 1 Department of Internal Medicine, Brody School of Medicine, East Carolina University, Greenville, NC 27834, USA; yangl@ecu.edu; Tel.: +1-252-744-3419; 2 Department of Anatomy and Cell Biology, Brody School of Medicine, East Carolina University, Greenville, NC 27834, USA

The tumor microenvironment has profound effects on cancer development, progression, and therapeutic response. Cancer cells co-exist with infiltrated immune cells, blood vessels, fibroblasts, and other stromal cells in the tumor microenvironment [1]. Deviated from their normal physiological roles, immune, vascular, and stromal cells in a tumor are often dysfunctional and exploited by cancer cells to promote tumor progression. Additionally, biochemical and biophysical characteristics of the tumor microenvironment are distinct from that in normal tissues. Several hallmarks of the tumor microenvironment, including hypoxia, acidosis, high interstitial fluid pressure, and increased extracellular matrix (ECM) stiffness, have been identified [2,3,4,5,6,7]. As initially proposed in the “seed and soil” hypothesis by Stephen Paget more than a century ago, tissue microenvironment is critical for cancer metastasis [8,9]. It has also been demonstrated that microenvironmental factors play important roles in multiple stages of cancer progression and in the modulation of cancer therapeutic responses [2,3,4,5,6,7]. Therapeutics targeting crucial components of the tumor microenvironment, such as blood vessels and immune cells, have been added to the arsenals of cancer therapy [1,3,10].

The metabolism of cancer cells is reprogrammed from that of normal cells. In the early twentieth century, Otto Warburg observed that cancer cells preferentially utilize glycolysis, instead of oxidative phosphorylation, for energy metabolism even in the presence of oxygen (now known as the “Warburg effect”) [11,12]. Glycolytic metabolism of glucose results in lactic acid, which can acidify the tumor microenvironment after being expelled by cancer cells [2,4,5]. Moreover, tumor-associated hypoxia can further increase cellular glycolysis as well as lactic acid production and accumulation in the tumor microenvironment due to rapid cancer growth and defective blood vessel perfusion. Acidosis and hypoxia are biochemical hallmarks of the tumor microenvironment and profoundly modulate cancer cell metabolism and disease progression [2,4,5]. In addition to glucose, cancer cells also utilize lipids, amino acids, and other substrates for metabolism and biosynthesis. Cancer cell metabolism has been exploited as a target for cancer treatment [6,13,14].

The purpose of the Special Issue “Tumor Microenvironment and Metabolism” published in the *International Journal of Molecular Sciences* is to illustrate some recent development in the field of tumor microenvironment and cancer cell metabolism. Overall research topics presented include the tumor microenvironment, hypoxia, acidosis, angiogenesis/anti-angiogenesis therapy, inflammation, chemokines/other cellular factors, metastasis, tumor immunity/cancer immunotherapy, glycolysis, lipid metabolism, and one-carbon metabolism of cancer cells.

In the issue, several papers discuss hypoxia, acidosis, and other chemico-physical aspects of the tumor microenvironment. The review by Koizume and Miyagi discusses tumor hypoxia and the formation of lipid droplets, a cellular organelle, as a mechanism to promote cancer cell survival in the microenvironment [15]. Deep and Schlaepfer review the effects of hypoxia on lipid metabolism in prostate cancer [16]. Bendinelli et al. discuss the roles of hypoxia in cancer bone metastasis, focusing on the molecular pathways of SPARC (secreted protein acidic and rich in cysteine), Runx2, Endothelin-1, HGF (hepatocyte growth factor), and HIF-1α (hypoxia inducible factor 1α) [17]. The research article by Maeda et al. reports that CD133, a cancer stem cell marker, regulates HIF-1α expression under hypoxia and promotes migration and epithelial mesenchymal transition (EMT) of pancreatic cancer cells [18]. In addition to the papers on hypoxia, the research article by Dong et al. describes the effects of acidosis on vascular endothelial cells and demonstrates that acidosis stimulates endoplasmic reticulum (ER) stress response pathways through the pH-sensing receptor GPR4 (G protein-coupled receptor 4) in endothelial cells [19]. The research article by Lin et al. reports that a reduction of mitochondrial DNA copy numbers in renal cancer cells results in decreased mitochondrial respiration, increased glycolysis, augmented cell invasion, and resistance to chemotherapeutic drug doxorubicin in vitro [20]. Russo et al. present findings to show that the ribosomal protein uL3 functions as a regulator of oxidative stress responses and can re-sensitize multidrug-resistant lung cancer cells to chemotherapeutics [21]. Moreover, biophysical aspects of the tumor microenvironment are also discussed. Wang et al. review the rigidity and structural stability of extracellular matrix in the tumor microenvironment, focusing on the roles of lysyl oxidase (LOX) in crosslinking collagen and elastin by oxidation, maintaining ECM rigidity and stability, and regulating cancer cell invasion and metastasis [22]. The research article by Riwaldt et al. describes the effects of microgravity on gene expression in spheroid formation of thyroid cancer cells and reveals the involvement of several pathways, such as VEGF (vascular endothelial growth factor), MMPs (matrix metalloproteinases), and actin, which regulate the rigidity of cytoskeletal proteins and the level of extracellular proteins [23].

Angiogenesis is critical for tumor growth and metastasis. However, blood vessels in a tumor are often structurally and functionally defective, which is a contributing factor to hypoxia and acidosis in the tumor microenvironment [1,3,5]. The review by Simone et al. summarizes the molecular pathways important for angiogenesis and discusses pre-clinical studies and clinical trials of anti-angiogenic drugs in biliary tract cancer [24]. Angiolini and Cavallaro review the general aspects of tumor angiogenesis and anti-angiogenic therapy in cancer and then emphasize the role of the adhesion molecule L1CAM (L1 cell adhesion molecule) in tumor angiogenesis [25]. Guo et al. report the role of mast cell tryptase (MCT) in pancreatic cancer angiogenesis and demonstrate that MCT stimulates angiogenesis through upregulating angiopoietin-1 expression and the MCT inhibitor nafamostat exhibits anti-angiogenic activities [26]. The research article by Cao et al. reveals that the transcription factor Runx2 promotes vasculogenic mimicry of hepatocellular carcinoma cells (HCC) and increases the EMT, migration, and invasion of HCC [27].

Tumors are abnormal organ-like structures comprised of multiple cell types and extracellular matrix [1,3]. Cell–cell interactions through autocrine and paracrine signals play important roles in tumor growth and metastasis. Itatani et al. discuss the role of chemokines in the invasion and metastasis of colorectal cancer, focusing on chemokines and their receptors expressed by cancer cells and bone marrow-derived cells (BMDC) recruited to the tumor microenvironment [28]. Ji reviews the involvement of chemokines and growth factors in lymph node metastasis of cancer [29]. The research article by Han et al. reports that tissue factor (TF) produced by lung cancer cells in the tumor microenvironment stimulates complement activation, myeloid-derived suppressor cell (MDSC) recruitment, and tumor growth in the xenograft mouse model [30]. Apart from soluble factors, Roma-Rodrigues et al. review the role of exosomes, a type of nanovesicles produced by cancer cells, in tumorigenesis and discuss emerging technologies to exploit gold nanoparticles targeting malicious exosomes for cancer therapy [31]. The research article by Xue et al. demonstrates that CAPS1 (calcium-dependent activator protein for secretion 1) inhibits tumor growth by modulating vesicle exocytosis of hepatocellular carcinoma (HCC) cells and loss of CAPS1 expression in HCC tissues is associated with poor overall survival of patients [32]. Moreover, Tahmasebi Birgani and Carloni use the tumor microenvironment of hepatocellular carcinoma as an example to review the roles of major signaling pathways and cancer-associated cellular components, such as inflammatory cells, vascular cells, and stromal cells, in tumorigenesis and cancer therapy [33]. Binnemars-Postma et al. focus on the biology of tumor-associated macrophages (TAM) and discuss current strategies of using nanoparticles to target TAM for therapeutic purpose [34]. Additionally, the research article by Visser et al. characterizes the immune cell components in the altered microenvironment of nodular lymphocyte predominant Hodgkin lymphoma (NLPHL), revealing decreases in CD20+ B cells and CD56+ natural killer cells and an increase in CD4+ T cells in NLPHL in comparison to reactive lymph nodes (RLN) [35]. The fundamental aspects of cancer immunotherapy have also been discussed. Recent development of immune checkpoint blockade against CTLA-4 and PD-1/PD-L1 has resulted in the improvement of overall survival in a subset of cancer patients [10]. Botti et al. discuss the heterogeneous expression of PD-L1 in solid tumors and emphasize on the importance of standardizing PD-L1 immunohistochemistry of tumor tissue microarrays as a prognostic biomarker [36]. Additionally, Du et al. discuss the need to rationally target multiple immune checkpoint pathways for cancer treatment, focusing on the immunoregulatory roles of TIM-3 (T cell immunoglobulin and mucin domain 3) [37].

As described earlier, aerobic glycolysis (the Warburg effect) is a common feature observed in many types of cancer cells [6,11,12,13]. Recent studies have also demonstrated that, in addition to glucose, cancer cells utilize multiple sources of substrates, such as lipids and amino acids, for energetic metabolism and some of the metabolic intermediates are also used by cancer cells for biosynthesis [6,13,14]. Deep and Schlaepfer discuss the aberrant lipid metabolism in prostate cancer, focusing on de novo lipid synthesis of cancer cells, lipid oxidation that supports cancer cell proliferation and survival, and the effects of hypoxia on lipid metabolism in prostate cancer [16]. The review by Moore and Pidgeon describes the role of bioactive lipids, 5-hydroxyeicosatetraenoic acid (5-HETE) and leukotrienes, and the 5-lipoxygenase enzyme in immune regulation, angiogenesis, as well as the pro- and anti-tumorigenic effects of the 5-lipoxygenase/bioactive lipid signaling pathways in cancer [38]. Koizume and Miyagi discuss the formation of lipid droplets in cancer cells as a mechanism for cancer cells to adapt to stress conditions, such as hypoxia, in the tumor microenvironment [15]. The research article by Notarnicola et al. reports that dietary ω-3 polyunsaturated fatty acids (ω-3 PUFAs) inhibits intestinal polyp growth in Apc^Min/+^ mice, which is correlated with the upregulation of cannabinoid receptor-1 (CB1) [39].

Additionally, cancer cells depend on one-carbon metabolism, such as the folate and methionine cycles, for the maintenance of DNA synthesis, epigenetic regulation, and redox homeostasis [40]. Corbin and Ruiz-Echevarría review the major pathways involved in one-carbon metabolism and discuss the role of androgen signaling in the regulation of one-carbon metabolism as well as DNA and histone epigenetic methylation in prostate cancer [41]. Parfett and Desaulniers describe the Tox21 approach to assess alterations of multiple epigenetic pathways in carcinogen-induced oncogenesis [42]. The research article by Lopomo et al. investigates the DNA methylation status of genes involved in one-carbon metabolism (MTHFR) and DNA methylation (DNMT1, DNMT3A, and DNMT3B) and reveals an increase of MTHFR promoter methylation in thymomas obtained from patients with myasthenia gravis [43]. Moreover, the research article by Wu et al. examines genetic polymorphisms related to one-carbon metabolism and vitamin B6 in a cohort of Chinese breast cancer patients, demonstrating that the polymorphisms of genes involved in one-carbon metabolism are associated with breast cancer susceptibility and that vitamin B6 deficiency can induce genomic instability in cultured cells [44].

In summary, the papers published in the Special Issue address multiple facets of tumor microenvironment and metabolism. The interesting topics discussed range from biomolecular aspects, such as hypoxia, acidosis, the Warburg effect, cancer cell metabolism, oxidative stress, extracellular matrix, chemokines, cytokines, growth factors, and signaling pathways, to cellular aspects, such as angiogenesis, immune cells, stromal cells, cancer cell growth, and metastasis. Chemotherapy and immunotherapy targeting the tumor microenvironment and metabolism have also been reviewed. We would like to thank all the authors for their contributions. The goal of the issue is to stimulate research and discussion in the growing field of tumor microenvironment and cancer cell metabolism. The importance of tumor microenvironment in oncogenesis is now increasingly recognized. Cancer cells do not exist in isolation; instead they co-exist with vascular cells, immune cells, and stromal cells in a distinct, complex microenvironment [1]. Cancer cell metabolism is also very distinct from the metabolism of normal cells [6,13]. Equally complex is the reciprocal crosstalk between cancer cells, stromal cells, and the tumor microenvironment. Co-evolution and adaptation of cancer cells to the tumor microenvironment play critical roles in regulating disease progression and therapeutic response. Recent research development prompts us to revisit the “seed and soil” hypothesis originally proposed by Stephen Paget more than a century ago [8]. Mounting evidence suggest that targeting both the “seed” (cancer cells) and the “soil” (tumor microenvironment) can be advantageous for cancer therapy [2,3,4,5,6,7]. The development of anti-angiogenesis therapy and cancer immunotherapy is a testament to this concept [1,3,7,10,45]. Despite significant research progresses, the complex interactions between cancer cells, metabolism, and the tumor microenvironment are not fully understood. For instance, multiplex inhibitory signals in the tumor microenvironment that hinder cancer immunotherapy are still ill-defined. Future research to better understand tumor microenvironment and cancer cell metabolism will prove to be beneficial for a comprehensive elucidation of tumor biology and the improvement of cancer therapy.
